# The Book World of Medicine and Science

**Published:** 1894-04-14

**Authors:** 


### The Book World of Medicine and Science.

### BOOK REVIEWS.

Common Neuroses. By James Frederic Goodhart, M.D., F.R.C.P. (Lewis, London.)

This little book is a valuable one, and we heartily recommend it to every thoughtful student of medicine. The truths it tells are familiar to all whose life-work lies amongst those individuals who belong to neurotic families, but it is written with such freshness and vigour of expression that one finishes its perusal with regret. Whether the type of certain diseases be altering or no, it cannot be disputed that our present mode of life lends itself to the development of the neurotic temperament, nor can it be doubted that the influence of the neurotic taint is becoming more potent. A long practice among those under this influence, and more especially if the majority of them be females, is apt to lead one to view with great suspicion any symptoms for which no organic cause can be found. Certainly there are many of such cases to whom the administration of medicines is a positive evil, and we hail with pleasure the author's bold and truthful words on this subject. The aim of the scientific physician should be the cure of his patient. In such cases the routine giving of drugs is not likely to attain that end, but rather to make the patient into a confirmed invalid. As the author says, the highest position the physician can take "is to cure people by advice rather than by drugs, to make the people pay for the use of our brains and not for a prescription of so many ounces of physic." He furthermore utters a warning which might with advantage be dinned into the ears of the profession at large that there is a time "when to give drugs is but quackery."

This is a view which in theory will be very generally received, yet there are difficulties in the way of putting it into practice. In the medical profession competition is severe, and the struggle for existence is often a hard one. Not only has the medical man to compete with his professional brother who is often not over scrupulous, but the prescribing chemist is now a formidable opponent. The doctor cannot afford to run the risk of losing patients. His neurotic patient rejects, with scorn, the plain spoken advice given where physic was expected. He was asked for bread and has offered a stone. If Jones at one end of the street will not order drugs, Brown at the other is ready and willing to do so. Jones is well aware of this fact, and with a family to provide for cannot afford to be a martyr for the truth. How can the public be expected to distinguish clearly between the relative worth of the doctor and the chemist when the aim of each is but to pocket a fee, and in return give a bottle of physic? We look forward to a time when, to quote the author's words, it will be recognized that a sound common sense, or perhaps, a robust physiology has a larger mission even than drugs in the treatment of disease. The latter, important though they be, are not everything. It is contrary to the spirit of modern medicine to think that the true remedy for every ill is "something out of a bottle."

Aero-Therapeutics. By C. Theodore Williams, M.D. (London: Macmillan and Co., 1894.)

This book consists almost entirely of matter which has been previously put before the medical profession either in the author's Lumleian lectures, in his well-known paper, read before the Royal Medico-Chirurgical Society, or in his address on the climate of Colorado. But it must not be thought that we think it on this account any the less valuable, for we strongly hold that the previous publications by Dr. Theodore Williams are so interesting that he has been quite right in collecting them together in the form of a book. There is no branch of therapeutic on which, as a rule, doctors show greater ignorance than the climatic treatment of disease, and we fear that in many cases the fatal event is actually hastened by injudicious advice given to the patient. It cannot be too strongly insisted that this department of therapeutics is, like any other, an art, and that a patient cannot be off-hand recommended to go abroad just because he happens to have phthisis. Each element must be considered in each case, and a valuable decision can only be given by the man who is not only skilful in diagnosis, but who knows the details of the climate of the various health resorts. Those who are ignorant on these matters had better at once read Dr. Theodore Williams' book, in which they will find a very good general account of the various factors of a climate which are of medical importance, a concise description of such places as are frequented by invalids, a sketch of the class of patient for which each health resort is favourable, and, what is even of more importance, the reader is warned what patients should not go abroad. Another very valuable point about the work is that it is always possible to form an opinion as to the accommodation at each place, although there is none of that contemptible advertisement of particular hotels which disfigures so many books on climate. With nearly all the places mentioned Dr. Williams gives a statistical account of the effects upon the patients whom he has sent there, and we are very glad to see that such good results can be obtained. They clearly show that it is of the very utmost importance for the cure of phthisis that it should be taken in hand early, but when this is done there is a very fair hope in the majority of cases that the sufferer may improve considerably, and sometimes the disease is actually arrested. The only parts of the book that seem to us unsatisfactory are those which discuss treatment by compressed and rarified air artificially prepared, and of the diseases induced by working under high atmospheric pressure; but this is not a matter of much importance, and the rest of the book is so very good that we can most cordially advise our readers to get it.

### THE PERIODICALS.

The Medical Magazine for this month is dedicated to Guido Baccelli, the President of the International Medical Congress, now being held in Rome, a compliment which the President reciprocates by contributing a short paper on the value of carbolic acid in neuralgia and tetanus. Quite apart from the present interest which attaches to its author, this article is of considerable scientific merit, and suggests a line of treatment for these intractable conditions which will be read with interest by all medical men. In the same issue there appears a paper by W. North on "Climate and Malaria," the outcome of certain researches carried on by the author in the malarial plains of Italy. It is a controversial essay, which will hardly appeal to most of the members of the Congress now sitting at Rome, and to whom opportunities are offered of observing the plasmodium malariæ under the microscope. The author of this paper ascribes the various symptoms of malaria as due not to a living organism, but to a disturbance of the central nervous system by the sudden changes of temperature, such as those to which the inhabitants of malarial districts are exposed. The theory has the merit of originality, but sadly lacks those objective and convincing proofs which appeal to the present day pathologist. There is also in this number an interesting review of Dr. Deneffe's "Chirurgie Antique," in which we are introduced to the instrumental stock in trade of a surgeon of the third century, and by which we are still further confirmed in the belief, at least as far as surgical instruments are concerned, that there is nothing new under the sun. Messrs. Macnamara and Stanley Boyd, in the educational section, submit their own views on the proposed Gresham University, as well as an abstract on the Commissioners' report, in two articles entitled respectively "A Teaching University for London" and "On the Report of the Gresham Commission."

